# Stereotactic Radiosurgery and Stereotactic Body Radiation Therapy Cost-Effectiveness Results

**DOI:** 10.3389/fonc.2013.00077

**Published:** 2013-04-08

**Authors:** Akash Bijlani, Giovanni Aguzzi, David W. Schaal, Pantaleo Romanelli

**Affiliations:** ^1^Accuray IncorporatedSunnyvale, CA, USA; ^2^CyberKnife CenterCentro Diagnostico Italiano, Milan, Italy

**Keywords:** cost-effectiveness, health economics, stereotactic body radiotherapy, stereotactic body radiation therapy, stereotactic radiosurgery, cancer

## Abstract

**Objective:** To describe and synthesize the current stereotactic radiosurgery (SRS) and stereotactic body radiation therapy (SBRT) cost-effectiveness research to date across several common SRS and SBRT applications.

**Methods:** This review was limited to comparative economic evaluations of SRS, SBRT, and alternative treatments (e.g., other radiotherapy techniques or surgery). Based on PubMed searches using the terms, “stereotactic,” “SRS,” “stereotactic radiotherapy,” “stereotactic body radiotherapy,” “SBRT,” “stereotactic ablative radiotherapy,” “economic evaluation,” “quality adjusted life year (QALY),” “cost,” “cost-effectiveness,” “cost-utility,” and “cost analysis,” published studies of cost-effectiveness and health economics were obtained. Included were articles in peer-reviewed journals that presented a comparison of costs between treatment alternatives from January 1997 to November 2012. Papers were excluded if they did not present cost calculations, therapeutic cost comparisons, or health economic endpoints.

**Results:** Clinical outcomes and costs of SRS and SBRT were compared to other therapies for treatment of cancer in the brain, spine, lung, prostate, and pancreas. Treatment outcomes for SRS and SBRT are usually superior or comparable, and cost-effective, relative to alternative techniques.

**Conclusion:** Based on the review of current SRS and SBRT clinical and health economic literature, from a patient perspective, SRS and SBRT provide patients a clinically effective treatment option, while from the payer and provider perspective, SRS and SBRT demonstrate cost savings.

## Introduction

For over 40 years clinicians have treated intracraniallesions with stereotactic radiosurgery (SRS). In the beginning, this non-invasive, highly innovative technique was received with great skepticism by the leaders of academic neurosurgery and radiation oncology. After 20 years of successful use in Sweden, the first Gamma Knife treatment was performed in North America in 1987 at the University of Pittsburgh. In 1989, the Gamma Knife was first used to treat the most common brain pathology, brain metastases (Lindquist, 1989). Since that time, radiosurgery has been shown to have excellent clinical outcomes and to be a cost-effective treatment option for patients with brain metastases (Lee et al., 2009).

Starting in 1995, Blomgren et al. (1995) and Hamilton et al. (1995) provided the first results of radiosurgical techniques outside of brain and spine in a procedure that has come to be called stereotactic body radiation therapy (SBRT). SBRT is the image-guided delivery of high dose radiation in an extremely hypofractionated treatment (typically up to five fractions). Delivering such high doses per fraction requires high conformality and steep dose fall-off to avoid irradiating organs at risk; this necessitates image-guidance for patient setup and, preferably, throughout treatment to adjust for changes in tumor/target position, thus minimizing treatment-related toxicity. Advances in image-guidance have allowed clinicians to safely deliver both SRS and SBRT, and as the growing body of literature has supported the safety and efficacy of these procedures, their utilization has steadily increased.

Before the advent of frameless techniques, Gamma Knife was primarily used to deliver SRS. Although it is an effective radiosurgery device, it is limited to treating only intracranial and upper spinal lesions due to the necessity of a rigid head frame. The frame also effectively limits the Gamma Knife to single-session (or fraction) treatment. For intracranial lesions, fractionated SRS may provide additional normal tissue protection when treating tumors near functional regions such as the optic chiasm or inner ear (Chang et al., 2005; Adler et al., 2006). The CyberKnife was built on the principles of Gamma Knife radiosurgery, delivering both isocentric treatments (like Gamma Knife) and non-isocentric treatments, but does not require a rigid head frame. Instead frequent image-guidance is used to locate the target (in some cases based on the position of implanted fiducial markers) and adjust the beam aim should any changes in target position be detected. The CyberKnife is also able to track and automatically correct for respiratory motion of targets in lung, liver, pancreas, etc. Although the CyberKnife was designed specifically for SRS/SBRT, SBRT, and SRS can also be delivered by gantry-based radiotherapy systems (e.g., Varian Truebeam, BrainLab’s Novalis TX, or BrainLab/Mitsubishi Vero). These systems also employ image-guidance for patient setup and, in some cases, occasional intra-fraction verification of target position, in addition to some combination of patient and target restraint using body frames and abdominal compression devices, breathing control, or respiratory gating to manage respiratory motion, and implanted fiducials or electromagnetic beacons.

As SRS and SBRT have grown, so has the clinical literature describing its application in treating a variety of tumors and lesions throughout the body including those in the brain, head/neck, spine, lung, liver, prostate, and pancreas. Despite the growing body of clinical SRS and SBRT literature, there is limited research into the cost-effectiveness and health economic outcomes of these procedures. Our long-term goal is to develop valid health economic research on SBRT and SRS; the current paper aims to describe and synthesize the SRS and SBRT cost-effectiveness research to date for several common SRS/SBRT indications.

## Methods

### Search strategy

Based on a PubMed search using the terms, “stereotactic,” “SRS,” “stereotactic radiotherapy,” “stereotactic body radiotherapy,” “SBRT,” “stereotactic ablative radiotherapy,” “economic evaluation,” “quality adjusted life year (QALY),” “cost,” “cost-effectiveness,” “cost-utility,” and “cost analysis,” published studies of cost-effectiveness and health economics were obtained. Inclusion criteria were limited to articles in published peer-reviewed journals and needed to include a comparison of costs between alternatives from January 1997 to November 2012.

### Inclusion/exclusion criteria

This review includes only comparative studies of SRS, SBRT, and alternative treatments in economic evaluations. Inclusion criteria were limited to articles in published peer-reviewed journals and needed to include a comparison of costs between alternatives from January 1997 to November 2012. Exclusion criteria included the absence of cost calculations, therapeutic cost comparisons, and health economic endpoints. Title, abstracts and full-text articles of all identified studies were reviewed independently by two co-authors.

## Brain

There are several published cost-effectiveness studies that focus on the clinical efficacy and cost-effectiveness of SRS compared to surgery (Table 1). One of the main reasons for this is that patients are treated with SRS on an outpatient basis compared with surgery, which requires utilization of inpatient hospital resources. Vuong et al. (2013) found that the average cost in Germany per patient for surgical resection was €11,647 compared to €9,964 for SRS. In addition, the survival time for surgical resection was 13.0 months while the survival time for SRS was 18.4 months. Also in Germany, Wellis et al. (2003) calculated the treatment costs of SRS and microsurgery for the treatment of meningiomas, acoustic neuromas, metastases, and arteriovenous malformations. For microsurgery, the average hospitalization time was 15.4 ± 8.6 days with 1.2 ± 2.8 of those days spent in the intensive care unit (ICU). The total average costs of microsurgery per patient including ancillary therapy and unplanned readmissions was €15,252, while the total average cost of SRS per patient was €7,920. Along the same lines, in Netherlands, van Roijen et al. (1997) analyzed costs and effects of treating acoustic neuroma patients with either microsurgery or radiosurgery. Direct costs for microsurgery were Dfl. 20,072 and Dfl. 14,272 for radiosurgery, while indirect costs were Dfl. 16,400 for microsurgery and Dfl. 1,020 for radiosurgery. In addition, the general health rating was better for radiosurgery than for microsurgery. Banerjee et al. (2008) also compared the costs of microsurgery to radiosurgery for the treatment of vestibular schwannoma. For microsurgery patients who were followed up for at least 36 months, mean surgical costs were $23,788, while for radiosurgery patients, the mean surgical costs were $16,143. For microsurgery patients, the mean follow-up costs per month started at over $1,000 per month and decreased steadily to less than $70 per month by the tenth month of follow-up. The mean follow-up costs for patients in the radiosurgery group were less than $10 per month for the first few months and thereafter increased to as much as $200 per month. In addition, the microsurgery patients suffered a significant decline from pre-operative levels in several components of the health status questionnaire (HSQ) at 3 months, 1 year, and most-recent follow-up; however, the radiosurgery group showed no decline in HSQ across all follow-up time frames.

**Table 1:** **Brain publication characteristics, estimated costs and effectiveness**.

Reference	Country	Type of study	Procedures compared	Perspective	Cost types	Local currency	Procedures cost per patient	Effectiveness	ICER/ICUR/Cost analysis results
Vuong et al. (2013)	Germany	Cost-effectiveness	SRS Surgery	Service provider	Direct	Euro	SRS: €9,964 Surgery: €11,647	SRS: 1.8 LY Surgery: 1.1 LY	SRS dominates
Wellis et al. (2003)	Germany	Cost analysis	SRS Surgery	Service provider	Direct	Euro	SRS: €7,920 Surgery: €15,242	n.a.	SRS is cost saving
van Roijen et al. (1997)	Netherlands	Cost analysis	SRS Surgery	Societal	Direct plus indirect	Dutch Florin	SRS: Dfl. 15,292 Surgery: Dfl. 36,472	n.a.	SRS is cost saving
Banerjee et al. (2008)	USA	Cost analysis	SRS Surgery	Service provider	Direct	USD	SRS: $16,143 Surgery: $23,788	n.a.	SRS is cost saving
Manning et al. (2000)	USA	Cost-effectiveness	SRS HSRT	Service provider	Direct	USD	HSRT was $4119 less costly than SRS	Median survival of 11.8 months from HSRT treatment	HSRT is more comfortable for patients and less costly than SRS
Cho et al. (2006)	Taiwan	Cost analysis	SRS Surgery	Societal	Direct plus indirect	USD	SRS: $15,881 Surgery: $44,130	n.a.	SRS is cost saving
Tarricone et al. (2008)	Italy	Cost-effectiveness	SRS Surgery	Service provider	Direct	Euro	SRS: €7,920 Surgery: €15,242	Not statistically significant difference in BNI score for SRS and Surgery at follow-up	SRS is cost saving
Mehta et al. (1997)	USA	Cost-utility	RT plus surgery	Service provider	Direct	USD	RT plus SRS: $15,102 RT plus Surgery: $22,018	RT plus SRS median survival: 1.1 LY RT plus surgery: 0.8 LY RT plus SRS median survival: 1.0 QALY RT plus surgery: 0.7 QALY	RT plus SRS dominates
Lal et al. (2012)	USA	Cost-utility	SRS plus observation SRS plus WBRT	Service provider	Direct	USD	SRS plus observation: $119,000 SRS plus WBRT: $74,000	SRS plus observation: 1.64 LY SRS plus WBRT: 0.6 LY ICER for SRS + observation: QALY (10); Effectiveness = 1.48; ICER = $41,783/QALY QALY (5); Effectiveness = 1.52; ICER = $43,280/QALY QALY (1); Effectiveness = 1.54; ICER = $44,064/QALY	SRS plus observation vs. SRS plus WBRT $44,231/LYS; $41,783/QALY
Rutigliano et al. (1995)	USA	Cost-effectiveness	SRS and surgery	Healthcare payer	Direct	USD	SRS: $22,743 Surgery: $30,461	SRS: $24,811/LY Surgery: $32,149/LY	SRS “has a better incremental cost–effectiveness than surgical resection (US$40,648 vs. 52,384 per life year)”
Tan et al. (2011)	Netherlands	Cost analysis	SRS RT Surgery	Service provider	Direct	Euro	SRS: €3,966 RT: €3,060 Surgery: €14,329	n.a.	SRS and RT are cost saving alternative compared to surgery

*RT, radiation therapy; SRS, stereotactic radiosurgery; HSRT, hypofractionated stereotactic radiotherapy; WBRT, whole-brain radiation therapy; USD, United States dollar; LY/LYS, life years/life year saved; QALY, quality adjusted life years; ICER, incremental cost-effectiveness ratio; ICUR, incremental cost-utility ratio; BNI score, barrow neurological institute pain intensity scoring criteria; n.a., not applicable*.

Manning et al. (2000) compared the treatment cost of linac-based hypofractionated stereotactic radiotherapy (HSRT) and SRS for the treatment of brain metastases. The median absolute cost of SRS was $4,119 higher than HSRT. In Taiwan, Cho et al. (2006) compared the direct and indirect costs from both hospital and societal perspectives for SRS and open surgery for the treatment of benign cranial base tumors. For open surgery, the mean length of stay was 18.2 ± 30.4 days including 5.0 ± 14.7 days of ICU stay and 13.0 ± 15.2 days of ward stay. The mean hospital stay for SRS was 2.2 ± 0.9 days with no need of ICU stay. The mean loss of workdays for open surgery was 160 ± 158 and 8.0 ± 9.0 days for SRS. The direct cost for SRS was higher than that for open surgery ($9,677 ± $6,700 vs. $5,837 ± 6,587). Open surgery had a higher complication rate (31.2%) compared to SRS (3.8%). Open surgery had a mortality rate of 5.3% while there was no mortality for SRS. The socioeconomic costs were significantly higher for open surgery compared to SRS ($34,453 ± 97,277 vs. $10,044 ± 7,481). Finally, the cost per QALY was significantly lower with SRS compared to open surgery ($3,762/QALY vs. $8,996/QALY). Along the same lines, Tarricone et al. (2008) compared the full treatment costs of SRS vs. microvascular decompression (MVD) for trigeminal neuralgia. The MVD full treatment costs were €6,641 per patient while the full SRS treatment costs were €4,388 per patient. The difference was attributed to the cost of the surgical procedure and the cost of inpatient hospitalization for MVD, which was, on average, 10 days (no hospitalization is required for SRS). Lal et al. (2012) utilized a decision analysis model to compare SRS plus observation vs. SRS plus whole-brain radiation therapy (WBRT). The median survival of the SRS plus observation group was 15.2 months, while the median survival for SRS plus WBRT was 5.7 months. However, the recurrence rates were higher for patients treated with SRS plus observation compared to SRS plus WBRT (71 vs. 15%). Compared with SRS plus WBRT, SRS plus observation had a higher average cost ($74,000 vs. $119,000) but a higher average effectiveness [0.60 life years saved (LYS) vs. 1.64, respectively] with an incremental cost-effectiveness ratio (ICER) of $44,231 per LYS or $41,783 per QALY (10-year horizon). Rutigliano et al. (1995) developed a cost-effectiveness model that compared the results of surgical resection and SRS for the treatment of solitary metastatic brain tumors. The study found that SRS had a lower uncomplicated procedure cost ($20,209 vs. $27,587), a lower average complication cost per case ($2,534 vs. $2,874), a lower total cost per procedure ($22,743 vs. $30,461), was more cost-effective ($24,811 vs. $32,149 per life year) and had a better incremental cost-effectiveness ($40,648 vs. $52,384 per life year) compared to surgical resection. Treatment-related morbidity and mortality were higher with surgical resection compared to radiosurgery (29.7 vs. 12.9%; 6.6 vs. 0%). Tan et al. (2011) compared the initial and post-treatment (1-year) costs of microsurgery, linac radiosurgery, and Gamma Knife radiosurgery in meningioma patients. Initial treatment costs were €12,299, €1,547, and €2,412 for microsurgery, linac radiosurgery, and Gamma Knife radiosurgery respectively. Microsurgery patients were admitted for an average of 11.3 inpatient days, which contributed to the higher microsurgery costs. Microsurgery inpatient stay cost was €5,321 while the indirect cost was €4,350. The microsurgery inpatient cost was nearly 14 times higher than linac or Gamma Knife radiosurgery (€5,321 vs. €386). In addition, the 1-year follow-up costs were €2,041 for microsurgery, €1,514 for linac radiosurgery, and €1,553 for Gamma Knife. This accounted for both treatment-related and treatment-unrelated costs. The annual total costs, including equipment cost per fraction, were €14,329 for microsurgery, €3,060 for linac radiosurgery, and €3,966 for Gamma Knife. Mehta et al. (1997) compared the outcomes of treatment with a combination therapy of radiation therapy (RT) plus surgery or RT plus radiosurgery. The median cost for RT plus surgery was $22,018 while the cost median costs of RT plus radiosurgery was $15,102, while the cost-effectiveness was significantly better for RT plus radiosurgery compared to RT plus surgery ($13,729 vs. $27,523 per year of survival gained). The average cost of QALY was $15,012 for RT plus radiosurgery, $31,454 per QALY for RT plus surgery, and $32,500 per QALY for RT alone.

Some of the limitations of these studies include the lack of direct clinical and health economic comparison between treatment options, resource cost utilization unrelated to treatment, as well as lack of following patient quality of life outcomes. Future cost-effectiveness study design should consider direct clinical and health economic comparisons between treatment options as well as capturing the follow-up costs related directly to treatment, and the cost of lost work-time and reduced efficiency. Although these studies reviewed do have some limitations, they are extremely valuable in demonstrating that as hospitals and health systems look to provide high-quality, cost-effective treatment options, compared to surgery, SRS is an attractive alternative.

## Spine

Although spine radiosurgery is a well-developed extracranial application of SRS and SBRT, and considerable efficacy and safety data have been published, there is limited data on the cost-effectiveness of the procedure (Table 2). In comparing external beam radiation therapy (EBRT) to SBRT for spinal metastases, Haley et al. (2011) found that the total cost to treat 100 patients with SBRT (including a 9% retreatment rate) was $842,420, while the cost to treat 30 Gy in 10 fractions (including a 23% retreatment rate) was $676,309 and the cost to treat 20 Gy in 5 fractions (including a 23% retreatment rate) was $499,911. As noted, although SBRT was more costly than EBRT, patients treated with EBRT had higher levels of acute toxicities and were more likely to require additional interventions at the treated sites. Papatheofanis et al. (2009) constructed a Markov model to simulate outcomes of patients undergoing non-chemotherapeutic interventions – either CyberKnife SRS or EBRT – for metastatic spinal tumors. Patients treated with CyberKnife SRS gained an additional net health benefit of 0.08 QALY while the CyberKnife SRS cost was $11,812 and EBRT was $13,745, a difference of $1,933.

**Table 2:** **Spine publication characteristics, estimated costs, and effectiveness**.

Reference	Country	Type of study	Procedures compared	Perspective	Cost types	Local currency	Procedures cost per patient	Effectiveness	ICER/ICUR/Cost analysis results
Haley et al. (2011)	USA	Cost-effectiveness	SBRT EBRT	Service provider	Direct	USD	SBRT: $7,729 EBRT: $5,498	No significant difference in overall survival	EBRT is cost saving
Papatheofanis et al. (2009)	USA	Cost-utility	SRS EBRT	Healthcare payer	Direct	USD	SBRT: $11,813 EBRT: $13,682	SBRT: 0.28 QALY EBRT: 0.20 QALY	SRS dominates

*SRS, stereotactic radiosurgery; SBRT, stereotactic body radiation therapy; EBRT, external beam radiation therapy; USD, United States dollar; QALY, quality adjusted life years; ICER, incremental cost-effectiveness ratio; ICUR, incremental cost-utility ratio*.

The main limitations of these studies were the lack of head-to-head comparative clinical and health economic data across therapy options and the fact that side effect treatments varied across patients.

Future trials should capture clinical and health economic data as well as quality of life indicators across all treatment options. The studies reviewed clearly demonstrate that SRS and SBRT provide clinicians with an additional cost-effective treatment option for spinal metastases that has better short-term results and comparable long-term results to EBRT.

## Lung

While surgical resection is the standard of care for many patients with non-small cell lung cancer (NSCLC), the location of the tumor and age and health status of patients with lung cancer often dictate whether they can undergo surgery. For those patients who are not surgical candidates, conventional RT and, more recently, SBRT, are treatment options. For many elderly patients with comorbid conditions such as emphysema and COPD, breath holding or controlled breathing (which may be required for RT delivered without tumor motion management capabilities) further reduces their options (Table 3). Lanni et al. (2011) compared the clinical and cost outcomes of SBRT, 3-dimensional conformal RT (3DCRT), and intensity modulated radiation therapy (IMRT) for the treatment of medically inoperable NSCLC. The treatment cost, calculated using the charge cost from the institution for the technical and professional components, for 35 fractions of 3DCRT was $55,705, $136,570 for 35 fractions of IMRT, and $52,471 for 4 fractions of SBRT. The actual cost for a 35-fraction 3DCRT ranged from $50,000 to $61,000, while the actual cost of a 4-fraction SBRT ranged from $41,000 to $57,000. At a median potential follow-up of up to 36 months, SBRT had higher overall survival compared to 3DCRT (71 vs. 42%). Sher et al. (2011) developed a Markov model comparing SBRT, 3DCRT, and radiofrequency ablation (RFA) for 65-year-old men with medically inoperable NSCLC. In the base-case analysis, RFA, 3DCRT, and SBRT had a mean cost per QALY of $44,648/1.45, $48,842/1.53, and $51,133/1.91, respectively. The ICER for SBRT over 3DCRT was $6,000/QALY and $14,100/QALY for SBRT over RFA. Compared to RFA and 3DCRT, SBRT had lower 3-year local recurrence, regional recurrence, and distant metastases rates. Puri et al. (2012) compared the cost-effectiveness of surgical intervention and SBRT in high-risk patients with stage I NSCLC. The median survival with surgery was 4.1 years, and the 4-year survival was 51.4%. With SBRT, the median survival was 2.9 years, and the 4-year survival was 30.1%. The cause-specific survival was identical between the two groups, and the difference in overall survival was not statistically significant. Nevertheless, SBRT was estimated to have a mean expected survival of 2.94 years at a cost of $14,153 and mean expected survival with surgery was 3.39 years at a cost of $17,629, for an ICER of $7,753.

**Table 3:** **Lung publication characteristics, estimated costs, and effectiveness**.

Reference	Country	Type of study	Procedures compared	Perspective	Cost types	Local currency	Procedures cost per patient	Effectiveness	ICER/ICUR/Cost analysis results
Lanni et al. (2011)	USA	Cost-effectiveness	SBRT 3DCRT IMRT	Service provider	Direct	USD	SBRT: $52,471 3DCRT: $55,705 IMRT: $136,570	SBRT 36-month overall survival: 71% 3DCRT 36-month overall survival: 42% IMRT 36-month overall survival: n.a.	SBRT dominates
Sher et al. (2011)	USA	Cost-utility	SBRT 3DCRT RFA	Service provider	Direct	USD	SBRT: $51,133 3DCRT: $48,842 RFA: $44,648	SBRT: 1.91 QALY 3DCRT: 1.53 QALY RFA: 1.45 QALY	SBRT vs. 3DCRT: $6,000/QALY SBRT vs. RFA: $14,100/QALY
Puri et al. (2012)	USA	Cost-effectiveness	SBRT Surgery	Healthcare payer	Direct	USD	SBRT: $14,153 Surgery: $17,629	SBRT overall survival: 2.94 years Surgery overall survival: 3.39 years	Surgery vs. SBRT: $7,753/LYS

*SBRT, stereotactic body radiation therapy; EBRT, external beam radiation therapy; 3DCRT, 3-dimensional conventional radiation therapy; IMRT, intensity modulated radiation therapy; RFA, radiofrequency ablation; USD, United States dollar; LY/LYS, life years/life year saved; QALY, quality adjusted life years; ICER, incremental cost-effectiveness ratio; ICUR, incremental cost-utility ratio*.

Limitations across these studies included the fact that the cost analysis was modeled from a Payer’s perspective, rather than a societal or combined perspective. In addition, since these studies were retrospective, survival benefits may not have been fully captured across all therapy options. Ongoing cost-effectiveness studies should be done prospectively and not only capture the clinical outcomes of the different treatment options, but also quality of life measures. Given the positive clinical and health economic outcomes, SBRT provides a cost-effective and clinically effective outpatient and non-invasive therapy option for patients with NSCLC compared to conventional RT and RFA, while surgery remains the first treatment option in terms of cost-effectiveness.

## Prostate

There are many different treatment options available to men diagnosed with localized prostate cancer including a variety of radiation therapies – 3DCRT, IMRT, proton therapy, SBRT, brachytherapy (HDR and LDR) – as well as surgical options – open, laparoscopic, and robotic (Table 4). Using a Markov model, Parthan et al. (2012) compared the cost-effectiveness of SBRT, IMRT, and proton therapy. The work-time lost due to treatment for SBRT, IMRT, and proton therapy was 10, 90, and 100 h, respectively. From a payer perspective, SBRT dominated both IMRT and proton therapy (SBRT: cost $24,873; QALY 8.11; IMRT: cost $33,068; QALY 8.05; proton therapy: cost $69,094; QALY 8.06). From a societal perspective, SBRT dominated both IMRT and proton therapy (SBRT: cost $25,097; QALY 8.11; IMRT: cost $35,088; QALY 8.05; proton therapy: cost $71,339; QALY 8.06). Hodges et al. (2012) also utilized a Markov model to compare the cost-effectiveness of SBRT and IMRT. The model assumed IMRT costs of $29,530 and SBRT costs of $14,315. Results showed that the mean cost and QALYs for SBRT and IMRT were $22,152 and 7.9 years and $35,431 and 7.9 years, respectively.

**Table 4:** **Prostate and pancreas publication characteristics, estimated costs, and effectiveness**.

Reference	Country	Publication year	Type of study	Procedures compared	Perspective	Cost types	Local currency	Procedures cost per patient	Effectiveness	ICER/ICUR/Cost analysis results
Parthan et al. (2012)	USA	2012	Cost-utility	SBRT IMRT PT	Healthcare Payer/Societal	Direct/Direct plus indirect	USD	Healthcare Payer: SBRT: $24,873; IMRT: $33,068; PT: $69,094 Societal: SBRT: $25,097; IMRT: $35,088; PT: $71,339	SBRT: 8.11 QALY IMRT: 8.05 QALY PT: 8.17 QALY	SBRT dominates from both payer and societal perspectives
Hodges et al. (2012)	USA	2012	Cost-utility	SBRT IMRT	Healthcare Payer	Direct	USD	SBRT: $22,152 IMRT: $35,431	SBRT: 7.9 QALY IMRT: 7.9 QALY	SBRT is cost saving
Murphy et al. (2012)	USA	2012	Cost-utility	Gemcitabine alone Gemcitabine plus RT Gemcitabine plus IMRT Gemcitabine plus SBRT	Healthcare Payer	Direct	USD	Gemcitabine alone: $42,900 Gemcitabine plus RT: $59,900 Gemcitabine plus IMRT: $69 Gemcitabine plus SBRT: $ 56,700	Gemcitabine alone: 0.581 QALY Gemcitabine plus RT: 0.714 QALY Gemcitabine plus IMRT: 0.721 QALY Gemcitabine plus SBRT: 0.778 QALY	Gem. plus SBRT vs. Gem. alone: $69,500/QALY Gem. plus RT vs. Gem. alone: $126,800/QALY Gem. plus IMRT vs. Gem. plus RT: $1,584,100/QALY Gem. plus SBRT dominates both Gem. plus RT and Gem. plus IMRT

*RT, radiation therapy; SBRT, stereotactic body radiation therapy; PT, proton therapy; IMRT, intensity modulated radiation therapy; Gem, gemcitabine; USD, United States dollar; QALY, quality adjusted life years; ICER, incremental cost-effectiveness ratio; ICUR, incremental cost-utility ratio*.

Some of the limitations of these two studies include the limited long-term SBRT data for localized prostate cancer, thus potentially causing the current study models to inaccurately estimate SBRT clinical values. Future studies should focus not only on acute and late toxicity and long-term (5+ year) biochemical disease-free survival, but also focus on including cost and quality of life measures.

Collectively, these studies demonstrated that SBRT is a cost saving treatment option for localized prostate cancer.

## Pancreas

Recent studies, including the Eastern Cooperative Oncology Group study E4201, demonstrated improved survival when chemotherapy is combined with RT for patients with pancreatic cancer (Table 4). Murphy et al. (2012) compared the cost-effectiveness of four different therapies – gemcitabine, gemcitabine plus conventional RT, gemcitabine plus IMRT, and gemcitabine plus SBRT. The base-case cost of gemcitabine alone, gemcitabine plus SBRT, gemcitabine plus RT, and gemcitabine plus IMRT was $42,900, $56,700, $59,900, and $69,500, respectively. Overall, SBRT increased life expectancy by 0.20 QALY at an increased cost of $13,700 compared with gemcitabine alone (ICER = $69,500 per QALY). In the base-case analysis, gemcitabine plus SBRT dominated the more costly and less effective options of gemcitabine plus RT and gemcitabine plus IMRT. The study concluded that IMRT exceeds what society considers cost-effective in the treatment of locally advanced pancreatic cancer.

A limitation of this study was that the Markov model was used to compare preliminary results from phase 3 clinical trials (gemcitabine and gemcitabine plus RT in E4201) with phase 2 clinical data (gemcitabine plus SBRT). In addition, the model assumed actual costs and quality of life outcomes about supportive care for patients with pancreatic cancer. Future research needs should continue to capture the clinical outcomes but also add quality of life and cost measures. This will allow researchers to combine the clinical and health economic results in future publications. Based on the data reviewed, chemotherapy plus SBRT increased life expectancy compared to gemcitabine alone at a cost potentially acceptable by today’s standards.

## Conclusion

In our review of the current state of the research, SRS and SBRT have clearly demonstrated their clinical value and economic sustainability, often in comparison to long-standing and well-accepted treatment options. From a patient perspective, SRS and SBRT provide a patient-friendly treatment option compared to other treatment options such as conventional RT, especially those who live in a rural setting or a great distance from treatment centers. SRS and SBRT also offer a treatment option that is non-invasive and can be completed in the outpatient setting, thus potentially freeing up valuable inpatient hospital resources as well as allowing patients to resume their normal daily activity as quickly as possible. Both from payer and societal perspectives, the clinical and cost-effectiveness of SRS and SBRT have been demonstrated to reduce health system utilization (medication, retreatment, etc.) and minimize indirect costs, thus saving payers additional financial resources, and reducing the strain on the workforce. In times of increasing resource constraint cost-effective and cost saving techniques could be crucial for healthcare systems in order to maintain their sustainability in the long run. Identifying such techniques will require continued coupling of robust clinical research with economic data.

## Conflict of Interest Statement

The authors declare that the research was conducted in the absence of any commercial or financial relationships that could be construed as a potential conflict of interest.
